# *Lactiplantibacillus plantarum* S1 as a Novel Dual-Functional Probiotic Strain for High-Efficiency Organoselenium Biotransformation in Functional Food Development

**DOI:** 10.3390/foods14111851

**Published:** 2025-05-22

**Authors:** Lin Yuan, Jianfeng Yuan, Chen Gao, Haoming Zhao, Chengye Wu, Zhong-Hua Yang

**Affiliations:** 1 Xingzhi College, Zhejiang Normal University, Jinhua 321100, Chinajf_yuan@zjnu.edu.cn (J.Y.);; 2 College of Chemistry and Chemical Engineering, Wuhan University of Science and Technology, Wuhan 430081, China

**Keywords:** *Lactiplantibacillus plantarum*, organoselenium, se-enriched probiotic, biotransformation

## Abstract

The microbial conversion of inorganic Se into bioactive organoselenium compounds represents a cutting-edge strategy for developing functional foods with enhanced nutritional value. This study introduces *Lactiplantibacillus plantarum* S1, a novel Se-enriched probiotic strain isolated from traditional Chinese sauerkraut, and systematically optimizes its capacity for selenite biotransformation. Critical fermentation parameters—including sodium selenite supplementation timing (2 μg/mL added at mid-log phase, 7 h post-inoculation), pH (5.0), and anaerobic cultivation duration (12 h)—were identified as key determinants of conversion efficiency. The optimized protocol achieved a 72.3% organoselenium conversion yield, producing 626.6 μg/g cellular organoselenium while maintaining probiotic viability (2.28 × 10^9^ CFU/mL). Se speciation analysis demonstrated that 78.51% of intracellular Se existed in organic forms, with protein-bound Se constituting the predominant fraction (85.33%), followed by polysaccharide-associated (6.42%) and nucleic acid-linked (3.38%) species. The strain’s dual functionality as both an efficient Se bioconverter and a resilient probiotic carrier highlights its potential for nutraceutical applications. These findings not only establish a robust bioprocess for Se-enriched probiotic production but also reveal mechanistic insights into preferential Se incorporation into protein matrices. This work bridges microbial Se metabolism research with scalable functional food innovation, offering a sustainable platform for developing Se-fortified products with dual health benefits.

## 1. Introduction

Selenium (Se), an essential trace element with a narrow therapeutic window (recommended daily intake: 50 μg; upper limit: 400 μg/d) [1], serves as a critical cofactor in selenoproteins that govern redox homeostasis, immune function, and oncoprevention, while directly inhibiting microbial pathogens [2,3]. Despite dietary sources including seafood and fortified products, Se deficiency remains a global health burden, implicated in Kashin–Beck disease, neurodegenerative disorders (Parkinson’s and Alzheimer’s diseases), thyroid dysfunction, and gastrointestinal pathologies [4,5].

The element’s bioactivity and toxicity are speciation-dependent. Organic Se species exhibit higher bioavailability and significantly reduced cytotoxicity compared to inorganic selenite/selenate, while demonstrating enhanced therapeutic efficacy in cancer chemoprevention [6,7]. Although inorganic Se remains economically viable for supplementation, its low assimilation efficiency (<10% bioavailability) and narrow safety margin (toxicity threshold: ≥900 μg/d) severely constrain clinical utility [8,9]. Biocatalytic conversion of inorganic Se to organoselenium metabolites addresses these limitations by reducing toxicity while improving bioaccessibility [10]. However, the scarcity of natural organic Se sources and prohibitive chemical synthesis costs demand sustainable bioproduction strategies.

Microbial biotransformation has emerged as an eco-efficient platform for Se speciation, synergizing elemental enrichment with probiotic functionality [11,12,13]. *Saccharomyces cerevisiae*, a GRAS (Generally Recognized As Safe) organism, achieves industrial-scale production of organic Se (1–4.5 mg/g dry biomass), with methyl selenocysteine (SeMCys) constituting 58% of Se species in selenized yeast [14,15]. Probiotic bacteria such as *Bifidobacterium longum* and *Lactobacillus* spp. further enhance value through dual-action mechanisms—combining Se bioconversion with antitumor activity, oxidative stress resilience, and gut microbiome modulation [16,17]. Notably, Se metabolism can fortify microbial robustness, as evidenced by improved bile salt tolerance in *Lactobacillus reuteri* and amplified anti-inflammatory capacity in *Bacillus subtilis* [18,19].

Among probiotics, *Lactiplantibacillus* spp. (reclassified from *Lactobacillus*) exhibit exceptional promise due to their acid tolerance, metabolic versatility, and capacity to biosynthesize bioactive organoselenium compounds, including selenoproteins and selenopolysaccharides [20,21]. Selenium-enriched *Lactiplantibacillus plantarum* has demonstrated excellent biological activity, particularly in the context of liver injury protection and other health benefits [22,23,24]. Strains isolated from fermented foods (e.g., kimchi and sauerkraut) demonstrate dual functionality—enhancing gut barrier integrity while modulating microbial communities [25]. These organisms reduce selenite to organoselenium via sulfur assimilation pathways, achieving up to 90% incorporation efficiency into biomolecules [26]. However, conversion kinetics vary dramatically across strains due to genomic heterogeneity and cultivation parameters [27,28]. There are some unresolved challenges that hinder industrial translation, such as strain limitations, optimization of conversion process, and determination of organoselenium specification [29].

This study addresses these bottlenecks through isolation of a novel *Lactiplantibacillus plantarum* strain isolated from selenium (Se)-rich ecological niches that could serve as a dual-functional probiotic capable of simultaneously enhancing organoselenium biotransformation efficacy and gastrointestinal health benefits. Here, we isolate and characterize *L. plantarum* S1 from traditional Northeast Chinese sauerkraut fermentation broth—a unique microbial reservoir under prolonged Se-enriched conditions. This work focuses on (1) quantifying its Se enrichment capacity under varying bioprocessing regimes, (2) optimizing high-density cultivation parameters to synergize biomass production with Se uptake, and (3) employing speciation analysis to resolve organoselenium profiles. By integrating microbial ecology with precision fermentation engineering, this work establishes a framework for developing a dual-functional probiotic that concurrently mitigates Se deficiency and enhances gastrointestinal health as a functional probiotic.

## 2. Materials and Methods

### 2.1. Reagents and Culture Media

Sodium selenite and 3,3′-diaminobenzidine (DAB, analytical grade) were procured from Shanghai Aladdin Biochemical Technology Co., Ltd. (Shanghai, China). Proteose peptone and yeast extract were obtained from ThermoFisher Scientific™ (Shanghai, China). Analytical-grade reagents including agar, glucose, toluene, EDTA, perchloric acid, nitric acid, hydrochloric acid, and Tween 80 were sourced from Sinopharm Chemical Reagent Co., Ltd. (Shanghai, China). Bacterial genomic DNA extraction was performed using the Magen HiPure Microbial DNA Kit purchased from Magen Biotech (Guangzhou, China). *Lactiplantibacillus plantarum* (formerly *Lactobacillus plantarum*) was isolated from fermented Northeast Chinese sauerkraut broth collected in Tieli County, Heilongjiang Province, China.

*L. plantarum* was cultivated in de Man–Rogosa–Sharpe (MRS) medium with the following composition (g/L): proteose peptone (10.0), beef extract (10.0), yeast extract (5.0), glucose (20.0), sodium acetate (5.0), ammonium citrate dibasic (2.0), K_2_HPO_4_ (2.0), MgSO_4_·7H_2_O (0.58), MnSO_4_·H_2_O (0.25), Tween 80 (1.0 mL), and CaCO_3_ (15.0). The medium was adjusted to pH 6.5 using 1 M NaOH/HCl, dissolved in deionized water, and then sterilized by autoclaving (121 °C, 15 psi, 30 min). For solid media, 20 g/L agar was incorporated prior to sterilization.

### 2.2. Strain Isolation and Purification

*L. plantarum* was isolated from the fermentation broth of naturally anaerobic-fermented sauerkraut produced in Se-rich regions of Northeast China. Serial dilutions (10^−1^ to 10^−6^) were prepared in sterile saline (0.9% NaCl, *w*/*v*), and 20 μL aliquots of each dilution were aseptically spread onto MRS agar plates supplemented with 15 g/L CaCO_3_. Negative control plates (sterile saline only) were processed in parallel to verify sterility and identify contamination risks. Plates were incubated anaerobically at 37 °C for 24–48 h. Primary screening targeted colonies exhibiting calcium dissolution halos (≥2 mm diameter), opaque white pigmentation, and a smooth, circular morphology (1–2 mm). The selected colonies were streaked onto fresh CaCO_3_-MRS agar using a sterile inoculating loop, followed by anaerobic incubation (37 °C, 24 h). The purification cycle was repeated 5–6 times until axenic cultures were confirmed by uniform colony morphology and Gram staining. Cryopreservation was performed in 20% (*v*/*v*) glycerol–MRS broth at −80 °C for long-term storage.

### 2.3. Strain Identification

Purified putative lactic acid-producing bacteria isolates obtained from Northeast Chinese sauerkraut were characterized through a polyphasic approach combining morphological, physiological, biochemical, and molecular methods [30,31]. Detailed physiological and biochemical experiments are provided in Supplementary Materials Section S1. Genomic DNA was extracted from 24 h MRS broth cultures using the Magen HiPure Microbial DNA Kit from Magen Biotech (Guangzhou, China). Near-full-length 16S rRNA gene sequences were amplified using universal primers 27F (5′-AGAGTTTGATCCTGGCTCAG-3′) and 1492R (5′-GGTTACCTTGTTACGACTT-3′), followed by Sanger sequencing at Sangon Biotech (Shanghai, China). Sequence homology was determined via BLASTn analysis against the NCBI GenBank database. Phylogenetic reconstruction was performed in MEGA 11 using the neighbor-joining algorithm with 1000 bootstrap replicates, incorporating reference strains exhibiting > 99% 16S rRNA gene sequence similarity.

### 2.4. High-Density Cultivation of L. plantarum S1

High-density cultivation of *L. plantarum* S1 is important to its industry production, which was achieved through sequential single-factor and orthogonal array optimization. The cultivation was performed in 250 mL Erlenmeyer flasks containing 150 mL medium under static batch conditions. An anaerobic environment was established by nitrogen gas (N_2_) sparging before inoculation, followed by sealed incubation at 37 °C without agitation. Carbon sources (glucose, maltose, sucrose, α-lactose, and soluble starch) were screened at concentrations of 0–100 g/L. Nitrogen sources were optimized by adjusting casein peptone-to-ammonium sulfate ratios (1:1–2:1) with a fixed total nitrogen content (25 g/L). Nutrient uptake was enhanced by supplementing Tween 80 (0–4 mL/L) and trace elements (MnSO_4_: 0–0.4 g/L; MgSO_4_: 0–1.2 g/L). Cultivation parameters—inoculum size (1–5%, *v*/*v*), temperature (20–40 °C), initial pH (5.0–7.0), and agitation speed (0–200 rpm)—were systematically optimized, with biomass monitored via biomass and viable cell counts after 24 h of incubation. The optimized system demonstrated a 1.8-fold increase in biomass compared to baseline conditions, validated through growth kinetics and nutrient consumption profiles. This strategy balances metabolic efficiency with scalability for industrial *L. plantarum* S1 bioprocessing.

### 2.5. Optimization of Se Enrichment Conditions for L. plantarum S1

*L. plantarum* S1 serves dual roles as a probiotic and organic Se producer through biotransformation of sodium selenite (Na_2_SeO_3_). The Se enrichment efficiency was systematically optimized by evaluating three factors, selenite content, supplementation timing, and pH, based on the upper optimized high-density cultivation results. Cultures (4% inoculum) were incubated anaerobically in MRS broth (30 °C) containing 0–20 μg/mL Na_2_SeO_3_ to determine maximum selenite tolerance. Organic Se yield and viable cell counts were quantified at 24 h intervals, followed by biomass collection via centrifugation (12,000× *g* 10 min, 4 °C). Temporal optimization involved supplementing the optimal selenite concentration at 0–13 h post-inoculation. Parallel experiments assessed initial pH effects (4.0–6.0) on bioconversion efficiency. The organic Se conversion rate (%) was calculated as (organic Se/total Se) × 100, with optimization criteria focused on maximizing the bioconversion rate. Microbial activity and growth kinetics were validated through growth curve analysis. This systematic optimization established a reliable process for producing Se-enriched probiotics with sustained viability and enhanced organic Se synthesis.

### 2.6. Analysis of Se Speciation in Se-Enriched L. plantarum S1

Se-enriched *L. plantarum* S1 was produced under optimized conditions: pH 5.0 MRS medium supplemented with 2 μg/mL Na_2_SeO_3_ during early log phase (7 h post-inoculation), followed by 12 h of anaerobic cultivation (30 °C). Biomass was harvested by centrifugation 12,000× *g* 10 min, 4 °C), washed thrice with deionized water, and then lyophilized. Three Se-containing fractions (selenoproteins, Se-polysaccharides, and Se-nucleic acids) were sequentially extracted using modified protocols [32,33]. Total Se content was determined by 3,3′-diaminobenzidine spectrophotometry.

For selenoprotein extraction, lyophilized cells (1.0 g) were ultrasonicated (400 W, 3 s on/5 s off, 20 min) in 2.759 mg/mL lysozyme solution [34]. The lysate was centrifuged (20,000× *g* 15 min, 4 °C), and the supernatant underwent ammonium sulfate precipitation (80% saturation, 12 h, 4 °C). The precipitated proteins were dissolved in 0.05 M Tris–HCl (pH 8.0), dialyzed against the same buffer (4 °C), and then analyzed for protein concentration and Se content.

To the Se–polysaccharide isolation, the cell homogenates (1 g in 10 mL 1.0 M NaOH, 4 °C, 4 h) were centrifuged (20,000× *g* 15 min, 4 °C). Supernatants were deproteinized using the Sevag method [35], followed by ethanol precipitation (75% *v*/*v*). Polysaccharides were collected by centrifugation, redissolved in deionized water, and then purified through three-stage dialysis (3.5 kDa membrane, 4 °C). Purified fractions were lyophilized and stored at −20 °C.

For Se–nucleic acid purification, bead mill homogenization with CTAB buffer (800 μL) was applied to lyse 1.0 g lyophilized biomass. After Sevag deproteinization [35], nucleic acids were precipitated by pH adjustment to 2.5 (HCl, 4 °C overnight) and collected by centrifugation (20,000× *g* 15 min, 4 °C). Purity was verified by UV–Vis spectrophotometry (A_260_/A_280_ = 1.8–2.0) [36].

### 2.7. Analytical Methods

#### 2.7.1. Viable Cell Enumeration

Viable bacterial counts were quantified via plate counting according to China National Standard GB4789.35-2016 [37]. Serial dilutions were plated in triplicate on MRS agar and incubated anaerobically at 30 °C for 48 h. Colonies (30–300 per plate) were counted and expressed as CFU/mL.

#### 2.7.2. Se Content Analysis

Total Se was determined by 3,3′-diaminobenzidine (DAB) spectrophotometry [38]. Surface-bound inorganic Se was removed by centrifugation (12,000× *g* 10 min), followed by dialysis (100 Da membrane, 24 h) to isolate organic Se. Acid digestion (HCl:HNO_3_ 1:1 *v*/*v*, cold digestion overnight, then heated until fuming) converted organic Se to inorganic form [33]. Digestates were analyzed via the DAB reaction: Samples (35 mL) were mixed with 1 mL 5% EDTA-2Na (pH 2.5) and reacted with 4 mL 0.5% DAB at 60 °C (30 min dark incubation). Absorbance at 420 nm quantified Se using Na_2_SeO_3_ standards.

#### 2.7.3. Biomacromolecule Quantification

Proteins were quantified by a Bradford assay with Coomassie Brilliant Blue G-250 [39]. Polysaccharides were quantified by the sulfuric acid–UV method at 315 nm [40]. Nucleic acids were quantified through UV absorbance at 260 nm with DNA molar absorptivity (ε = 50 μg/mL/AU) [41].

#### 2.7.4. Statistical Analysis

All experiments were performed in triplicate. The data represent the mean of triplicate values. The corresponding standard deviation was calculated. All statistical analyses were conducted using Excel (2021) and OriginPro (2021).

## 3. Results and Discussion

### 3.1. Strain Isolation and Taxonomic Identification

**Figure 1:**
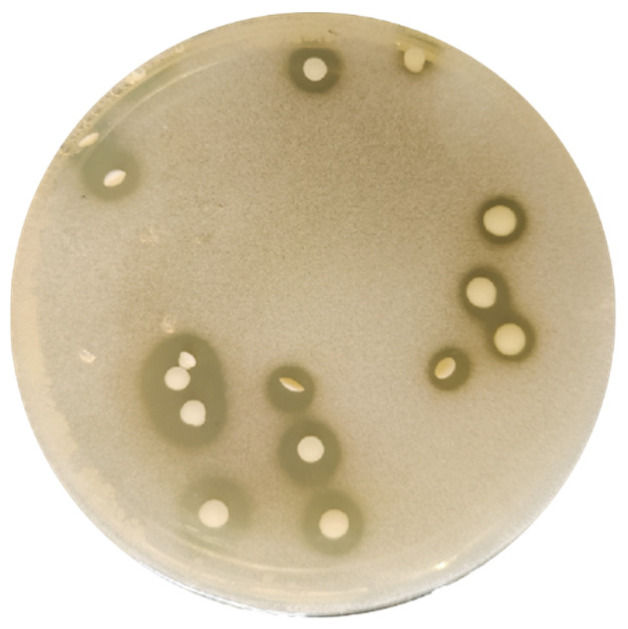
Purification plate of strains isolated from the fermentation broth of Northeast Chinese sauerkraut.

Target strain isolation was conducted through calcium dissolution halo screening (Figure 1) from Northeast Chinese sauerkraut fermentation broth. The traditional fermentation system of Northeast Chinese sauerkraut is known to harbor a diverse population of probiotics, particularly Lactobacillus species. Moreover, this region is rich in selenium; thus, it is promising to screen selenium-enriched lactic acid-producing probiotics from such indigenous fermentation systems [42,43]. Lactic acid-producing bacteria form distinct calcium solubilization halos on calcium carbonate-supplemented media due to their ability to convert insoluble calcium carbonate into soluble calcium ions. The diameter of the halo correlates with the strain’s lactic acid production capacity. Serial dilutions (10^−4^–10^−5^) were plated on MRS agar under microaerophilic conditions, with five successive purification cycles yielding axenic cultures. The 10^−5^ dilution demonstrated optimal isolation efficiency, producing 20 ± 3 CFU/plate with characteristic dissolution halos (4.2 ± 0.3 mm radius), indicative of organic acid production capacity.

**Figure 2:**
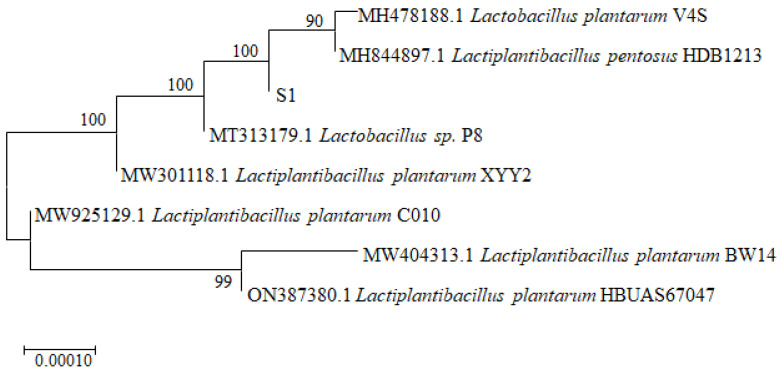
Phylogenetic tree of strain S1.

Phenotypic characterization revealed typical *Lactiplantibacillus* features through Gram staining (Gram-positive rods), catalase negativity, and carbohydrate fermentation profiles [30,31,44]. Detailed results can be found in the Supplementary Materials Section S1. Molecular identification via 16S rDNA sequencing Sangon Biotech (Shanghai) Co.,Ltd, Shanghai, China) generated a 1471 bp sequence (Supplementary Materials Figure S1) showing 100% homology with *Lactiplantibacillus plantarum* references in NCBI database. Phylogenetic analysis (Figure 2) confirmed closest relationship with *L. plantarum* V4S (MH478188.1), supported by a 99.8% bootstrap value. The strain was formally designated *Lactiplantibacillus plantarum* S1 as a potentially probiotic strain.

As a well-established probiotic [21], *L. plantarum* demonstrates exceptional Se biotransformation capacity. Recent studies highlight its Se-enriched derivatives’ therapeutic potential, including hepatoprotective effects and insulin regulation [22,23,24], warranting further investigation of strain S1′s functional properties.

### 3.2. Optimization and Performance Evaluation of L. plantarum S1 High-Density Culture

High-density cultivation (HDC) of *L. plantarum* S1 is critical for its industrial application as a Se-enriched probiotic. This work systematically optimized media composition and fermentation parameters to achieve its maximum viable bacteria counts and biomass yield, with full experimental datasets provided in the Supplementary Materials. These advancements establish a robust framework for scalable, cost-effective production of Se-fortified probiotics, addressing growing demand in the nutraceutical and fermented food sectors.

Carbon source screening identified sucrose (30 g/L) as optimal (Figure S2), yielding 1.25 × 10^9^ CFU/mL, outperforming glucose, maltose, and others. Sucrose’s low osmotic pressure and efficient metabolization by *Lactobacillus* spp., consistent with prior reports, minimized acid accumulation while sustaining growth. For nitrogen sources (Figure S3), yeast extract (16 g/L) combined with tryptone (8 g/L) achieved 1.29 × 10^9^ CFU/mL, leveraging synergistic amino acid and growth factor supply. Inorganic nitrogen sources, such as ammonium sulfate, proved inferior due to limited bioavailability. Tween 80 supplementation (1.5 mL/L) enhanced viable bacteria counts (Figure S4) by improving membrane fluidity and nutrient dispersion, though excessive concentrations (>1.5 mL/L) induced cytotoxicity. Trace element optimization highlighted Mg^2+^ (0.8 g/L MgSO_4_) and Mn^2+^ (0.2 g/L MnSO_4_) as critical enzyme cofactors, with higher doses disrupting metabolic balance (Figure S5).

**Figure 3:**
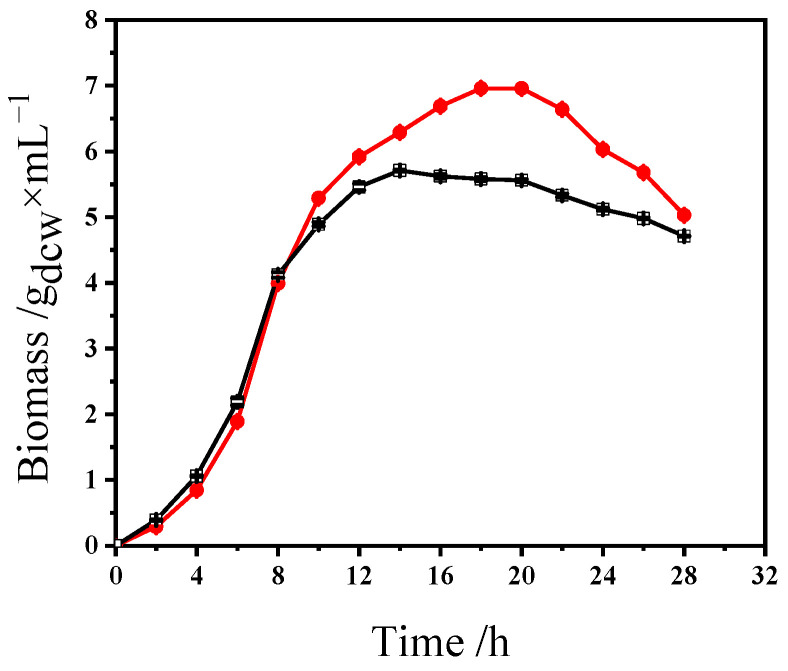
Growth curves of *Lactobacillus plantarum* S1 with and without optimized culture conditions. ●: with optimized culture conditions; □: without optimized culture conditions.

Fermentation parameters—initial pH, temperature, inoculum size, and shaker speed—were systematically optimized to work with the upper optimized media composition. An initial pH of 5.5 minimized acid stress during lactic acid synthesis, diverging from neutral pH optima reported for other *Lactobacillus* strains but critical for maintaining cell viability in prolonged fermentations [45]. A cultivation temperature of 30 °C maximized viable bacteria counts, achieving 1.33 × 10^9^ CFU/mL. Inoculum size optimization (4%) ensured balanced nutrient utilization and mitigated inhibitory byproduct accumulation, a key consideration for batch consistency in large-scale production [26]. Notably, static conditions (0 rpm) achieved 1.34 × 10^9^ CFU/mL, congruent with *L. plantarum* S1′s microaerophilic metabolism, whereas mechanical agitation (>50 rpm) caused shear stress-induced cell damage. *L. plantarum* is a facultative homolactic acid bacterium capable of efficient anaerobic fermentation. Static cultivation conditions reduce oxygen diffusion, thereby promoting anaerobic metabolism. Moreover, *L. plantarum* can form biofilms through the secretion of extracellular polysaccharides (EPSs) and adhesins, such as mannose-specific adhesins, which enhance local nutrient concentration and confer resistance to environmental stress. Static culture promotes biofilm development and, in turn, supports the proliferation of *L. plantarum*. The integrated optimal parameters (30 °C, 0 rpm, pH 5.8, 4% inoculum size) elevated viable bacteria counts to 1.49 × 10^9^ CFU/mL, representing a 16% increase over baseline conditions. Growth curves analysis, presented in Figure 3, revealed an extended logarithmic phase (4–12 h) and delayed stationary phase (20 h), indicative of sustained metabolic activity under optimized parameters—a critical advantage for industrial bioreactor throughput.

The sucrose preference aligns with *L. plantarum* CXG-4 studies, though optimal concentrations here (30 g/L) were lower than reported for some strains (40–50 g/L), likely due to strain-specific acid tolerance [26]. The yeast extract–tryptone synergy mirrors findings for *L. plantarum*, emphasizing organic nitrogen superiority [46]. Static cultivation contrasts with studies advocating mild agitation (50–100 rpm) for aerobic probiotics but suits *L. plantarum*’s microaerophilic metabolism. The final cell density (1.49 × 10^9^ CFU/mL) exceeds typical yields for non-optimized *L. plantarum* cultures (about 10^8^ CFU/mL) and rivals results from fed-batch systems, underscoring the cost-effectiveness of single-batch HDC. In conclusion, tailored media and culture parameters significantly enhance *L. plantarum* S1 biomass, providing a scalable framework for functional probiotic production.

### 3.3. Conversion of Inoranic to Organic Se by Se-Enriched L. plantarum S1

This study establishes a bioprocess engineering strategy to maximize the metabolic conversion of toxic selenite (Na_2_SeO_3_) into functional organic Se compounds, addressing two critical challenges in microbial Se enrichment: (1) balancing detoxification pathways with bioactive Se synthesis, and (2) maintaining probiotic viability under Se stress. Through systematic optimization, we achieved unprecedented organic Se yields (>73%) with enhanced biomass production, enabling cost-effective manufacturing of Se-fortified probiotics for nutritional intervention in Se-deficient populations.

**Figure 4:**
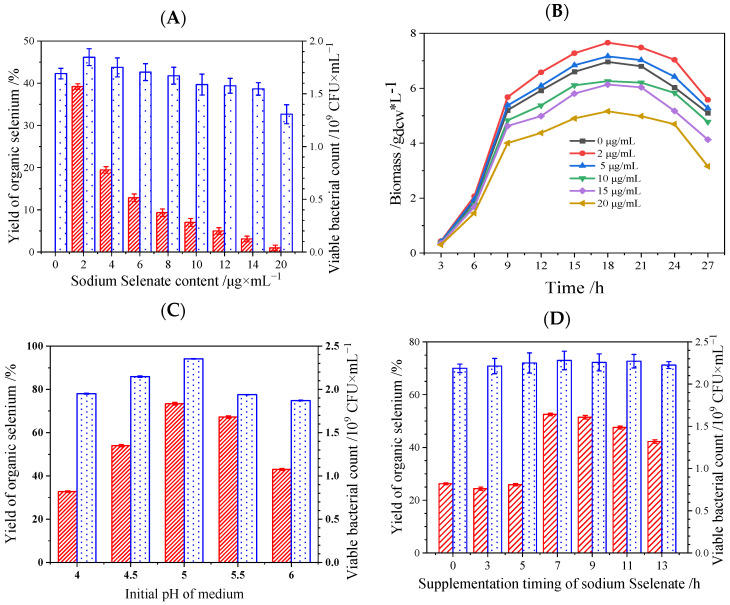
Effect of various factors on the conversion of inorganic Se to organic Se by *L. plantarum.* (**A**): Effect of sodium selenite content on organic Se yield and viable bacteria count; (**B**): growth curves of *L. plantarum* S1 under different Se contents; (**C**): organic Se yield and viable bacteria count of *L. plantarum* S1 under different initial pH media; (**D**): effect of sodium selenite addition time on organic Se yield and viable bacteria count. 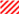: Yield of organic Se; 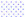: viable bacterial count.

Through extensive multi-factor investigation, the optimized selenium enriched culture conditions were obtained (Figure 4). Optimal selenite concentration (2 μg/mL) yielded 39.21% organic Se and 1.85 × 10^9^ CFU/mL (Figure 4A), leveraging the strain’s Se-dependent antioxidant system (glutathione peroxidase and thioredoxin reductase) to mitigate oxidative damage [26,47]. Elevated concentrations (≥4 μg/mL) triggered metabolic redirection toward elemental Se (Se^0^) precipitation—a detoxification mechanism conserved across *Bifidobacterium* and *Saccharomyces* [48,49]. Growth kinetics confirmed maximal biomass accumulation at 18 h under optimal selenite exposure (Figure 4B), contrasting with very obvious growth inhibition at 10 μg/mL.

Acidic cultivation (pH 5.0) maximized organic Se yield (73.4%) and viable counts (2.35 × 10^9^ CFU/mL) through dual mechanisms (Figure 4C): (1) protonation of surface carboxyl groups (-COO⁻→-COOH) enhanced selenite (SeO_3_^2−^) adsorption via electrostatic attraction, and (2) stabilization of selenite reductase activity (optimal pH 4.8–5.2) minimized enzyme denaturation [49]. Neutral/alkaline conditions (pH > 6.0) reduced conversion efficiency by 62% due to charge repulsion and enzyme instability.

Mid-log phase supplementation (7 h post-inoculation) achieved peak organic Se production (52.5% yield) and viable bacteria counts (2.28 × 10^9^ CFU/mL) (Figure 4D). This aligns with peak membrane transporter activity and selenocysteine synthase expression during exponential growth in *Lactobacillus* and *Bifidobacterium* spp. [48]. Early-phase addition (0 h) induced premature oxidative stress (23% viability loss), while late-phase supplementation (≥12 h) occurred during metabolic decline (ATP depletion and 58% lower NADPH availability) [49].

The integrated protocol (2 μg/mL Na_2_SeO_3_, pH 5.0, 7 h supplementation) enhanced organic Se yield to 73.4% and viable counts to 2.35 × 10^9^ CFU/mL. This represents the highest reported organic Se conversion efficiency for *Lactiplantibacillus* spp. *Enterococcus durans* and yeast, surpassing previous records (58–64%) [26,33,50]. The developed strategy provides a universal framework for microbial Se enrichment, with direct applications in functional foods and nutraceuticals targeting oxidative stress-related disorders (Type II diabetes and non-alcoholic fatty liver disease) [22]. *L. plantarum* S1 demonstrated superior performance in both organic Se conversion efficiency and tolerance to selenite stress. Furthermore, its high viable cell count (2.35 × 10⁹ CFU/mL under optimal conditions) highlights its potential as a dual-function probiotic–Se delivery system, outperforming many commercial *L. plantarum* strains used in functional foods.

### 3.4. Se Specification in Se-Enriched L. plantarum S1

This study quantitatively resolved the Se speciation profile of *L. plantarum* S1 to evaluate its functional superiority as a Se-enriched probiotic. With total Se content reaching 713.8 μg/g with 87.8% (626.6 μg/g) as bioactive organic forms, the strain demonstrates unprecedented Se biofortification capability, addressing critical gaps in microbial Se delivery systems for nutritional supplementation. The organic Se content is remarkably higher than the reported probiotics (from 35 to 207.51 μg/g) [48].

**Figure 5:**
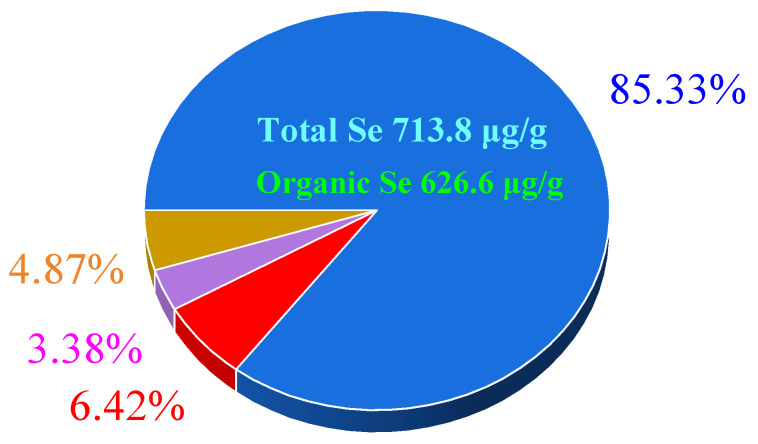
Proportion of various forms of organic Se. ■: Selenoprotein, ■: selenopolysaccharide, ■: se-associated nucleic acids, ■: unidentified forms.

Selenoproteins constituted 85.33% of organic Se (534.67 μg/g) (Figure 5), significantly exceeding values reported for *L. plantarum* CXG-4 (65.44%) and *Enterococcus durans* (56.3%) [26,33]. This high selenoprotein yield correlates with enhanced antioxidant enzyme activity, crucial for probiotic stress resistance and host health benefits. Se–polysaccharides (40.22 μg/g, 6.42%) and Se–nucleic acids (21.18 μg/g, 3.38%) showed species-specific variations, aligning with *L. plantarum* CXG-4′s polysaccharide profile but differing markedly from *E. durans* (35.2% Se-polysaccharides). Residual unidentified Se (4.87%) likely represents novel Se metabolites or cell wall complexes, warranting further characterization.

## 4. Conclusions

This study successfully isolated *L. plantarum* S1 from Se-rich Northeast Chinese sauerkraut fermentation broth, demonstrating dual functionality as a probiotic and high-efficiency Se biotransformer. The developed high-density cultivation protocol achieved 2.35 × 10^9^ CFU/mL with 73.4% organic Se conversion efficiency (626.6 μg/g). Selenoproteins dominated the speciation profile (85.33%), indicating robust selenocysteine (Sec) biosynthesis pathway activity—a key step in producing bioavailable and antioxidant-rich Se compounds.

The strain’s exceptional Se assimilation capacity (87.8% organic forms) addresses two global health challenges: (1) Se deficiency affecting 1 billion people worldwide due to geochemically poor soils, and (2) limited bioavailability (<30%) of synthetic Se supplements. By leveraging microbial biotransformation, this work provides a natural, food-grade solution delivering 62 μg bioavailable Se in just 0.1 g biomass—equivalent to 0.5 mg of commercial yeast-based supplements.

Despite these promising findings, several limitations should be acknowledged. First, the current study focused primarily on in vitro optimization of Se enrichment and probiotic performance; in vivo validation of Se bioavailability, antioxidant activity, and probiotic functionality remains lacking. Second, while preliminary stability assessments suggest that the organic Se forms are relatively stable under simulated storage conditions, further studies are needed to evaluate long-term Se retention during extended shelf life and under various processing conditions (e.g., freeze-drying, heat treatment, and refrigeration).

This microbial platform bridges the gap between nutritional science and food biotechnology, offering a sustainable strategy to combat Se malnutrition through culturally accepted fermented foods. However, for practical applications in functional food development, future research should focus on evaluating the behavior of Se-enriched *L. plantarum* S1 in real food matrices and assessing its performance in animal or human clinical trials. Additionally, mechanistic studies on the regulation of Se metabolism and Sec biosynthesis pathways in *L. plantarum* S1 could further enhance our understanding and improve bioprocessing strategies.

In summary, *L. plantarum* S1 represents a promising candidate for developing next-generation Se-fortified probiotics. With further optimization, *L. plantarum* S1 could catalyze a paradigm shift from pharmaceutical supplementation to dietary prevention of Se-deficiency disorders.

## Data Availability

The original contributions presented in the study are included in the article/Supplementary Materials. Further inquiries can be directed to the corresponding author.

## Supplementary Materials

The following supporting information can be downloaded at https://www.mdpi.com/article/10.3390/foods14111851/s1: Section S1: Physiological and Biochemical Characterization; Section S2: Molecular Identification via 16S rDNA Sequencing; Section S3: High-Density Cultivation of Se-Enriched *L. plantarum* S1; Table S1: Physiological and biochemical characteristics of strain S1; Figure S1: 16S rDNA sequence of strain S1; Figure S2: Selection of the most suitable carbon source; Figure S3: Selection of the most suitable nitrogen source; Figure S4: Optimization of Tween 80 amount; Figure S5: Optimization of magnesium ions and manganese ions; Figure S6: Optimization of fermentation parameters. Reference [30] is cited in the Supplementary Materials.
