# Supplementary material for: Lactiplantibacillus plantarum S1 as a Novel Dual-Functional Probiotic Strain for High-Efficiency Organoselenium Biotransformation in Functional Food Development
Source: Foods. 2025 May 22;14(11):1851. doi: 10.3390/foods14111851 (PMC12154060; doi:10.3390/foods14111851)
Supplement: Supplementary file 1 [file foods-14-01851-s001.zip › foods-3621992-supplementary.pdf]

Supplementary Materials

Section S1. Physiological and Biochemical Characterization

Physiological and biochemical assays were performed according to established protocols for *Lactobacillus* identification. Key results are summarized in STable 1:

- **Catalase activity**: Negative (no bubble formation), confirming the absence of catalase-mediated H_2O_2 decomposition.
- **Nitrate reduction and arginine hydrolysis**: Negative for both tests.
- **Carbohydrate fermentation**: BTB-MR assays demonstrated acid production in PY medium supplemented with glucose, xylose, lactose, sucrose, arabinose, maltose, soluble starch, inositol, and mannitol (positive reactions). Notably, **rhamnose** fermentation was negative, as evidenced by retained green coloration (no acid production).

These results fully align with the biochemical profile of *Lactiplantibacillus plantarum* described in *Common Bacterial System Identification Manual* [30].

Table S1. Physiological and biochemical characteristics of strain S1

Test	Result	Carbohydrate	Result
Catalase	–	Glucose	+
Gelatin liquefaction	–	Xylose	+
H₂S production	–	Lactose	+
Nitrate reduction	–	Sucrose	+
Voges-Proskauer (VP)	–	Arabinose	+
Starch hydrolysis	–	Maltose	+
Arginine deamination	–	Rhamnose	–
		Soluble starch	+
		Inositol	+
		Mannitol	+

(“+” = positive; “–” = negative)

Section S2. Molecular Identification via 16S rDNA Sequencing

For definitive classification, the 16S rDNA sequence of strain S1 (1471 bp; SFig. 1) was analyzed. BLAST alignment revealed **100% homology** with *Lactiplantibacillus plantarum* strains (e.g., *L. plantarum* V4S, MH478188.1). Phylogenetic analysis (MEGA 10.1.7) placed strain S1 within a clade containing validated *L. plantarum* strains.

```
1       CAGGACGAAC GCTGGCGGCG TGCCTAATAC ATGCAAGTCG AACGAACTCT
51      GGTATTGATT GGTGCTTGCA TCATGATTTA CATTTGAGTG AGTGGCGAAC
101     TGGTGAGTAA CACGTGGGAA ACCTGCCCAG AAGCGGGGGA TAACACCTGG
151     AAACAGATGC TAATACCGCA TAACAACTTG GACCGCATGG TCCGAGTTTG
201     AAAGATGGCT TCGGCTATCA CTTTTGGATG GTCCCGCGGC GTATTAGCTA
251     GATGGTGGGG TAACGGCTCA CCATGGCAAT GATACGTAGC CGACCTGAGA
301     GGGTAATCGG CCACATTGGG ACTGAGACAC GGCCCAAACT CCTACGGGAG
351     GCAGCAGTAG GGAATCTTCC ACAATGGACG AAAGTCTGAT GGAGCAACGC
401     CGCGTGAGTG AAGAAGGGTT TCGGCTCGTA AAACTCTGTT GTTAAAGAAG
451     AACATATCTG AGAGTAACTG TTCAGGTATT GACGGTATTT AACCAGAAAG
501     CCACGGCTAA CTACGTGCCA GCAGCCGCGG TAATACGTAG GTGGCAAGCG
551     TTGTCCGGAT TTATTGGGCG TAAAGCGAGC GCAGGCGGTT TTTTAAGTCT
601     GATGTGAAAG CCTTCGGCTC AACCGAAGAA GTGCATCGGA AACTGGGAAA
651     CTTGAGTGCA GAAGAGGACA GTGGAACTCC ATGTGTAGCG GTGAAATGCG
701     TAGATATATG GAAGAACACC AGTGGCGAAG GCGGCTGTCT GGTCTGTAAC
751     TGACGCTGAG GCTCGAAAGT ATGGGTAGCA AACAGGATTA GATACCCTGG
801     TAGTCCATAC CGTAAACGAT GAATGCTAAG TGTTGGAGGG TTTCCGCCCT
851     TCAGTGCTGC AGCTAACGCA TTAAGCATTC CGCCTGGGGA GTACGGCCGC
901     AAGGCTGAAA CTCAAAGGAA TTGACGGGGG CCCGCACAAG CGGTGGAGCA
951     TGTGGTTTAA TTCGAAGCTA CGCGAAGAAC CTTACCAGGT CTTGACATAC
1001    TATGCAAATC TAAGAGATTA GACGTTCCCT TCGGGGACAT GGATACAGGT
1051    GGTGCATGGT TGTCGTCAGC TCGTGTCGTG AGATGTTGGG TTAAGTCCCG
1101    CAACGAGCGC AACCCTTATT ATCAGTTGCC AGCATTAAGT TGGGCACTCT
1151    GGTGAGACTG CCGGTGACAA ACCGGAGGAA GGTGGGGATG ACGTCAAATC
1201    ATCATGCCCC TTATGACCTG GGCTACACAC GTGCTACAAT GGATGGTACA
1251    ACGAGTTGCG AACTCGCGAG AGTAAGCTAA TCTCTTAAAG CCATTCTCAG
1301    TTCGGATTGT AGGCTGCAAC TCGCCTACAT GAAGTCGGAA TCGCTAGTAA
1351    TCGCGGATCA GCATGCCGCG GTGAATACGT TCCCGGGCCT TGTACACACC
1401    GCCCGTCACA CCATGAGAGT TTGTAACACC CAAAGTCGGT GGGGTAACCT
1451    TTTAGGAACC AGCCGCCTAA G
```

Figure S1. 16S rDNA sequence of strain S1.

Section S3. High-Density Cultivation of Se-Enriched *L. plantarum* S1

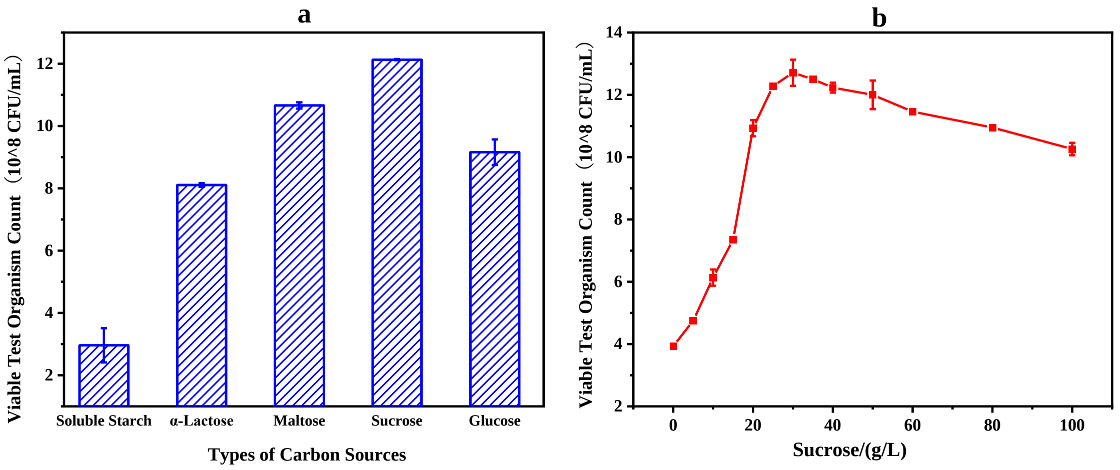

Figure S2. Selection of the most suitable carbon source

(a) Types of carbon sources; (b) Sucrose

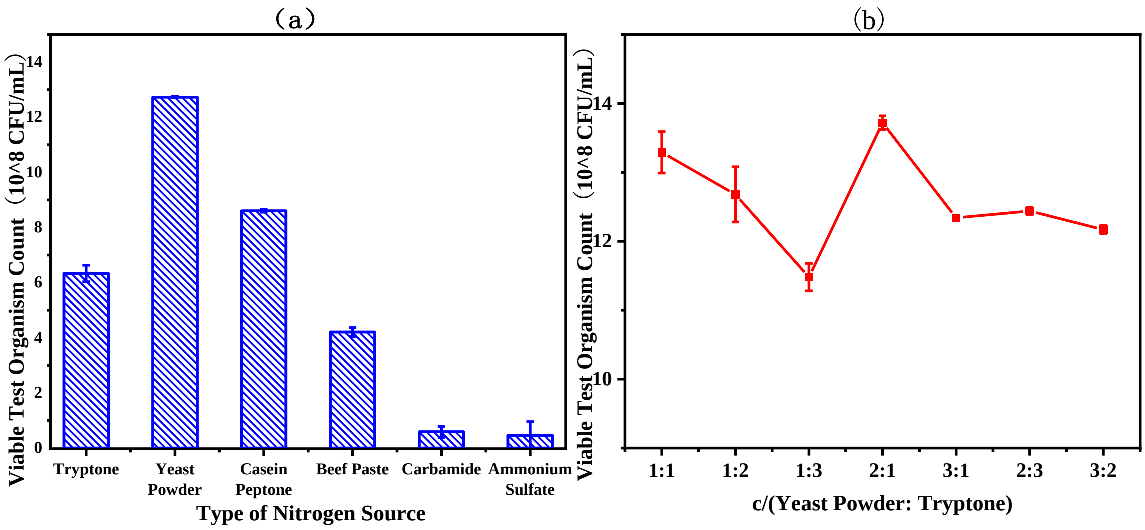

Figure S3. Selection of the most suitable nitrogen source

(a) Type of nitrogen source; (b) c/(Yeast Powder:Tryptone)

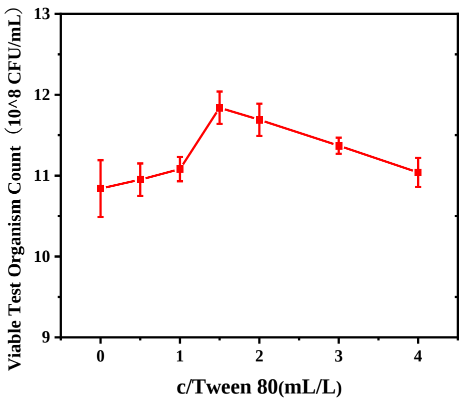

Figure S4. Optimization of Tween 80 amount

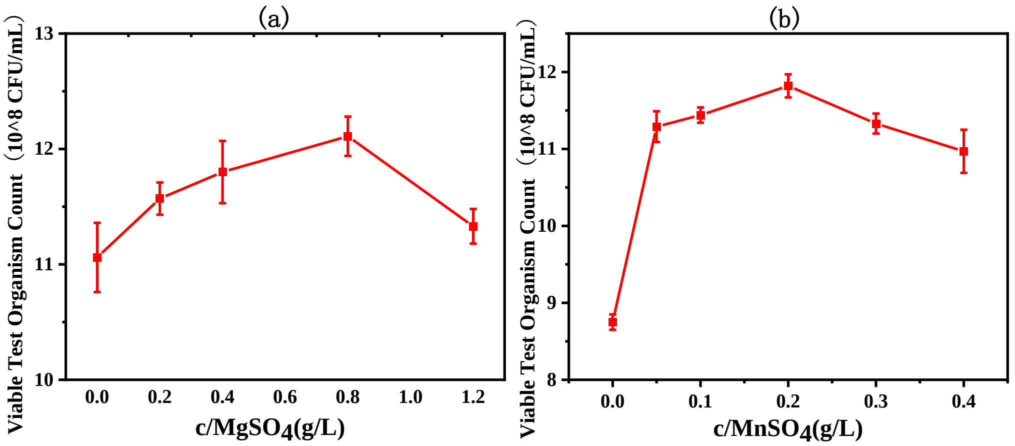

Figure S5. Optimization of magnesium ions and manganese ions

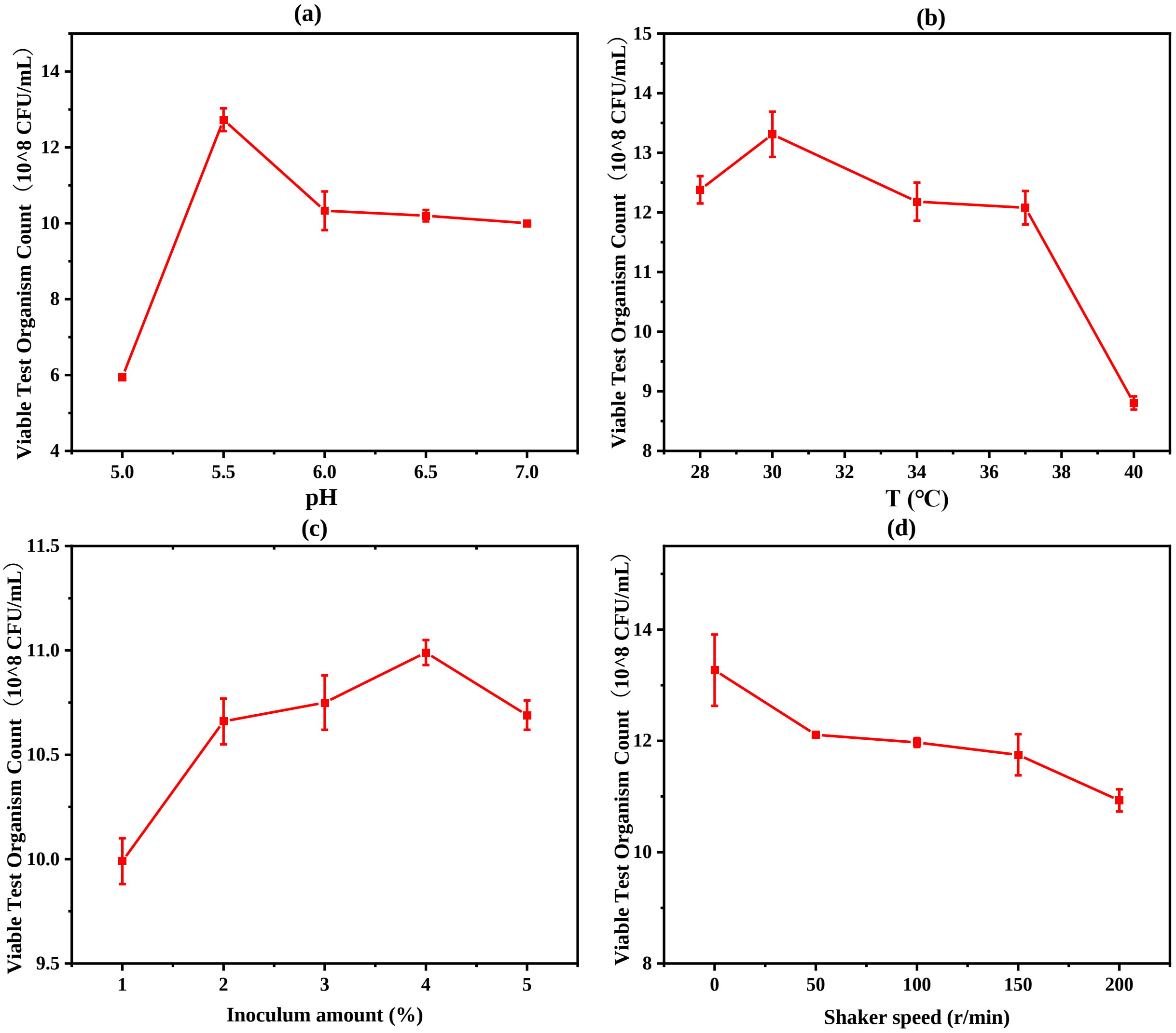

Figure S6 Optimization of fermentation parameters

(a): Initial pH value, (b): Cultivation temperature, (c): Inoculation amount, (d): Shaker speed
